# Seizure in a Seven-Year-Old With Hypertensive Encephalopathy: A Report of a Rare Case

**DOI:** 10.7759/cureus.92980

**Published:** 2025-09-22

**Authors:** Doa J Mirza, Mohammed R Hossain, Mirza Farooq Baig

**Affiliations:** 1 Emergency Medicine, Al Qassimi Hospital, Sharjah, ARE; 2 Emergency Medicine, Kuwait Hospital Sharjah, Sharjah, ARE; 3 Pediatric Emergency, Al Qassimi Women’s and Children’s Hospital, Sharjah, ARE

**Keywords:** hypertensive encephalopathy, pediatric emergency department, pediatric hypertensive emergency, pediatric seizure, pers

## Abstract

Hypertensive encephalopathy is a rare etiology for seizures in the pediatric population. A previously healthy seven-year-old boy presented to our Emergency Department (ED) with status epilepticus and high blood pressure. In spite of normal initial findings, the patient’s elevated blood pressure prompted concern for hypertensive encephalopathy. A subsequent magnetic resonance imaging (MRI) scan identified posterior reversible encephalopathy syndrome (PRES) as a condition resulting from uncontrolled high blood pressure. Following appropriate antihypertensive management, the patient’s symptoms were alleviated, and he was eventually discharged. The case highlights why hypertensive encephalopathy must be recognized as a potential cause of seizures in pediatric patients. It also shows the significance of measuring blood pressure in children as part of their initial assessment.

## Introduction

A seizure is a sudden episode of uncontrollable, involuntary motor, sensory, or autonomic events caused by abnormal and excessive electrical activity in the brain, and contributes to roughly 1% of all pediatric emergency department (ED) cases [1,2]. The most frequent causes of seizures are febrile illnesses, infections, hypoglycemia, and traumatic brain injury [3]. This report describes a seven-year-old boy with hypertensive encephalopathy who presented with seizures. Hypertensive encephalopathy is a severe complication of uncontrolled hypertension, distinguished by acute brain dysfunction resulting from elevated blood pressure [4]. It is a manifestation of hypertensive emergency, defined by a significant elevation in blood pressure, along with evidence of end-organ damage - in this case, the brain [5]. According to the American Academy of Pediatrics (AAP) guidelines, a significant elevation in blood pressure in children is defined as a reading above the 99th percentile for their age, sex, and height [6]. 

## Case presentation

In August 2024, a previously healthy seven-year-old male presented to the ED with a seizure lasting approximately 30 minutes, according to his caregiver. The patient reportedly experienced a generalized tonic-clonic seizure during sleep, accompanied by a staring look. He also had a history of low-grade tactile fever and rhinorrhea over the past two days, along with two episodes of vomiting during this time. There was no history of headache, visual disturbances, neck stiffness, or rashes.

He had no significant past medical history, except for a previous ED visit two months earlier for a headache. During that visit, he described a spontaneous, non-traumatic, central headache, associated with one episode of vomiting. He denied any prior similar episodes. His vital signs were stable, with a heart rate of 94 beats per minute and blood pressure of 125/64 mmHg, corresponding to a systolic blood pressure in the 94th percentile and diastolic blood pressure in the 50th percentile, based on the child’s age, sex, and height, according to the 2017 AAP Clinical Practice Guidelines for pediatric hypertension [7]. On examination, the patient was alert, with no neck stiffness or focal neurological deficits, and had a Glasgow Coma Scale (GCS) score of 15. Neurological, respiratory, cardiac, and abdominal examinations were unremarkable. His tympanic temperature was 37.3°C. The headache improved significantly with pain management using ibuprofen and paracetamol, and he was subsequently discharged.

Coming back to his current presentation, jerking movements were observed in the right upper and lower limbs, along with a fixed right-sided gaze. The patient appeared drowsy, was not obeying commands, and withdrew all limbs equally. Both pupils were sluggishly reactive. Reflexes were difficult to elicit, and there were no signs of meningeal irritation, as indicated by negative Kernig’s and Brudzinski’s signs. The patient measured 133 cm in height and weighed 36 kg. He was afebrile, with a temperature of 37.5°C, and had a blood pressure of 168/127 mmHg, corresponding to above the 99th percentile for systolic and diastolic pressures based on AAP guidelines. His heart rate was 125 beats per minute. Oxygen saturation was 97% on room air, and the respiratory rate was 23. Chest examination revealed poor respiratory effort with reduced bilateral air entry. The abdomen was soft and lax, with no tenderness or masses. Kernig’s and Brudzinski’s signs remained absent.

Based on the history and physical examination, differential diagnoses included hypertensive encephalopathy, electrolyte imbalances, hypoglycemia, hyperglycemia, meningitis, and febrile seizure.

Emergency room (ER) management

The seizure had been ongoing for approximately 30 minutes prior to arrival. On arrival, the patient was actively seizing and received 5 mg of rectal diazepam; however, the seizure persisted for a further 10 minutes. Subsequently, 2 mg of intravenous (IV) midazolam was administered, which successfully terminated the seizure. The patient was then placed in the recovery position, and oral secretions were suctioned. 

He was loaded with 1 g of IV levetiracetam (40 mg/kg). Initial venous blood gases (VBGs) were collected immediately, showing respiratory acidosis with pH 7.14, pCO_2_ 74.2 mmHg, pO_2_ 55.7 mmHg, HCO_3_^-^ 24.7 mmol/L, base excess -6.1 mmol/L, glucose 10.7 mmol/L, and lactate 3.3 mmol/L. During the next 30 minutes, the patient received IV fluids (0.9% sodium chloride, 340 mL, 10-20 mL/kg), was monitored in the recovery position, and stabilized post-ictally. A repeat VBG was then obtained, showing improvement: pH 7.27, pCO_2_ 59.3 mmHg, pO_2_ 63.8 mmHg, HCO_3_^- ^26.0 mmol/L, base excess -1.3 mmol/L, and lactate 1.7 mmol/L (Table 1).

**Table 1:** Initial and repeat VBG report VBG, venous blood gas

Parameters	Initial VBG	Repeat VBG (post-ictal)
pH	7.14	7.27
pCO_2_ (mmHg)	74.2	59.3
pO_2_ (mmHg)	55.7	63.8
HCO_3_^- ^(mmol/L)	24.7	26.0
Base Excess (mmol/L)	-6.1	-1.3
Glucose (mmol/L)	10.7	-
Lactate (mmol/L)	3.3	1.7

Post-ictally, the patient was drowsy, responsive to pain, withdrawing, and nearly localizing to pain, with incomprehensible sounds. His GCS was initially 8, with Eye (E) 1, Verbal (V) 2, and Motor (M) 5. Over a 90-minute post-ictal period, his GCS improved to 13 (E4, V3, and M6), eventually returning to 15 (E4, V5, and M6) with full consciousness.

A workup for a new-onset afebrile seizure was initiated. As part of the standard workup for status epilepticus, blood cultures and a comprehensive blood panel, including complete blood count, C-reactive protein, electrolytes, liver and renal function tests, and coagulation profile, were obtained to rule out infectious and metabolic causes. No bacterial growth was observed on blood cultures collected on day 1 and day 3, and all other laboratory findings were insignificant. Additionally, a non-contrast computed tomography (CT) scan of the head, conducted in the ER, was unremarkable. 

However, throughout the emergency stay, the patient's blood pressure had consecutively high readings of 170/130 and other readings in a similar range. Despite changing the cuff and machine, the blood pressure remained high. The highest measured reading was 182/132, following which IV hydralazine 0.1 mg/kg bolus was given. Initial hypertensive management temporarily dropped the blood pressure to 169/130 but then spiked back to 189/131, prompting the addition of IV labetalol 0.1 mg/kg/hr. Moreover, an electrocardiogram (ECG) and chest X-ray were obtained. The ECG showed normal sinus tachycardia with no significant changes, and the chest X-ray was unremarkable. 

**Figure 1:**
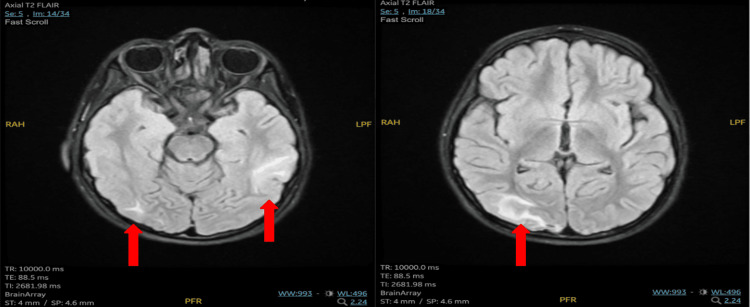
Axial T2 FLAIR MRI showing cortical/subcortical hyperintense signal in the right occipital and left temporo-occipital regions (arrows) MRI, magnetic resonance imaging; FLAIR, fluid-attenuated inversion recovery

The patient was stabilized and admitted to the Pediatric Intensive Care Unit (PICU), where a multidisciplinary team was consulted. A comprehensive diagnostic workup was conducted, including a lumbar puncture to rule out meningitis, which was negative. The results of a thorough assessment helped exclude alternative causes, making hypertensive encephalopathy the most likely diagnosis. Plain and contrast magnetic resonance imaging (MRI) revealed right occipital and left temporo-occipital cortical/subcortical areas of hyperintense T2/fluid-attenuated inversion recovery (FLAIR) signal, causing effacement of the overlying cortical sulci. It was not associated with diffusion restriction or post-contrast enhancement (Figure 1). These MRI findings, as well as clinical presentation, are highly indicative of posterior reversible encephalopathy syndrome (PRES), which is a subsequent complication of hypertensive encephalopathy.

Moreover, ophthalmological examination and transthoracic echocardiography were conducted to further assess potential complications of hypertension. The ophthalmological examination was unremarkable. However, the echocardiography revealed hypertensive heart disease with reduced left ventricular systolic function (ejection fraction of 42%), a dilated left ventricle, mild mitral regurgitation, no coarctation of the aorta, and pulmonary artery systolic pressure of 30 mmHg (Table 2). These echocardiographic findings provided evidence of hypertensive cardiomyopathy. No echocardiographic images were available; however, the report has been attached (Table 2).

**Table 2:** Transthoracic echocardiogram report NZ, normal zone

Cardiac Function	
Left ventricular posterior wall end-diastole (LVPWED)	6 mm NZ
Septum end-diastole (ED)	7 mm NZ
Septum end-systole (ES)	8 mm NZ
Left ventricular end-diastole (LVED)	40 mm NZ
Left ventricular end-systole (LVES)	32 mm (+2 Z score)
Left ventricular ejection fraction percentage (LV EF%)	42%
Left atrium/Aortic root (LA/Ao)	1.2

Further investigations were done to ascertain the underlying cause of hypertension, which included conducting an ultrasound of the kidney, ureters, and bladder (KUB); Doppler studies of the renal arteries; urinalysis; and a complete hormonal evaluation, including 24-hour urine catecholamine testing and assessment of plasma renin and aldosterone levels. 

Elevated plasma aldosterone and renin levels were found, which confirmed the diagnosis of secondary hyperaldosteronism. Upon administering appropriate management, the patient's blood pressure showed signs of improvement. The patient was eventually discharged with a proper follow-up plan. 

On follow-up, no underlying cause was identified on radioisotope scanning of the kidneys, and further investigations were deferred, as he remained asymptomatic. Subsequent blood pressure readings were within the normal range, and antihypertensive medications were not required. He is being followed up in the Neurology clinic due to his prior episode of hypertensive encephalopathy and the abnormal electroencephalogram (EEG) findings, and he remains on a maintenance dose of levetiracetam for seizure prophylaxis.

## Discussion

Hypertensive emergency 

Pediatric hypertension is defined as systolic and/or diastolic blood pressure readings at or above the 95th percentile for a child’s age, sex, and height on at least three different occasions [7]. Moreover, a hypertensive crisis refers to a sudden and severe elevation in blood pressure. While there is no fixed threshold to define severe hypertension, the AAP recommends that healthcare personnel be alert and on the lookout for possible end-organ damage when a pediatric patient’s blood pressure increases by 30 mmHg or more above the 95th percentile for their age and height [6,8]. 

Hypertensive crises are further divided into two categories: hypertensive urgency and hypertensive emergency. Hypertensive urgency refers to a severe and sudden rise in blood pressure without any evidence of end-organ damage, while hypertensive emergency involves a severe rise in blood pressure with signs of end-organ damage [5].

Hypertensive emergencies are relatively rare in children, occurring in approximately 2 out of every 10,000 visits to the ED [8]. Data acquired from the National Health and Nutrition Examination Survey (NHANES) in preadolescent and adolescent patients showed that the incidence of hypertensive crisis ranges between 1% and 4% [9]. Furthermore, retrospective studies in ED settings have reported a prevalence of hypertensive crisis among hypertensive patients ranging from 16% to 54% [10,11].

Measuring blood pressure in children is an imperative part of medical evaluation when they present to the ED. According to some authors, ED visits may provide the only occasion for hypertension screening [12]. Furthermore, it can be challenging to obtain follow-up for abnormal blood pressure measurements derived from initial screenings. A retrospective study has shown that although blood pressure was elevated in 52% of the pediatric patients who underwent triage blood pressure measurement, only 38% of those patients had repeated measurements [13].

As for the clinical presentation of hypertensive emergency, it differs by age in the pediatric population. For instance, infants and younger children typically present with non-specific symptoms such as failure to thrive, poor feeding, and irritability. On the other hand, older children who can narrate their symptoms may report headaches, visual disturbances, chest pain, dizziness, nausea, or vomiting. The complications attributed to hypertensive emergency involve the cardiac, renal, and neurological systems [5]. In our case, hypertensive encephalopathy was observed in the patient, which is defined as acute brain dysfunction resulting in headache, retinal hemorrhage, changes in consciousness, and seizures - due to elevated blood pressure [14].

While seizures are a common presentation in pediatric ERs, hypertensive encephalopathy as a cause of seizures is exceedingly rare, particularly in children. Furthermore, as mentioned earlier, hypertensive emergencies are also uncommon, with a prevalence of approximately 2 out of every 10,000 visits to the ED [8]. With hypertensive emergencies being a cause of hypertensive encephalopathy leading to seizures [5], the case underscores the importance of considering rare but critical differential diagnoses in pediatric seizures.

Moreover, a complication of hypertensive encephalopathy is PRES, which was also strongly suggested by the MRI findings. The condition can present with symptoms such as altered mentation, drowsiness, visual disturbances, seizures, and headaches. PRES may present either acutely or subacutely, with symptoms developing within hours to days [15]. 

Management of hypertensive emergency

When it comes to the management of hypertensive emergencies, it is recommended to lower the blood pressure to (i) below the 95th percentile in children with hypertension but no evidence of end-organ damage, and (ii) below the 90th percentile in children with end-organ damage [6]. An approach to blood pressure management described by Flynn and Tullus recommends reducing blood pressure by 25% of the target reduction within the first 8-12 hours, another 25% in the subsequent 8-12 hours, and the remaining 50% over the next 24 hours [16].

Management is generally carried out via IV access for administering antihypertensive medications, fluids, and other necessary treatments. IV medications are preferred for initial management, as they are fast-acting. However, in situations where IV access is challenging, or in cases of less severe blood pressure elevations, oral medications may be used. Among the various antihypertensives available, only hydralazine (IV and oral), sodium nitroprusside, minoxidil, and fenoldopam have been approved for use in pediatric patients by the United States Food and Drug Administration (FDA) [5].

Initial visit

Was a Further Extensive Workup Necessary During the Initial Visit?

According to the National Institute for Health and Care Excellence (NICE) guidelines, there is an emphasis on a selective and individualized approach to investigating headaches in children, guided by the presence of risk factors and red flags [17]. This approach suggests that routine investigations are generally not necessary in the absence of concerning features. 

In our patient, the initial presentation consisted of a single episode of central headache, associated with one episode of vomiting, followed by a normal neurological examination and improvement of symptoms with simple analgesics such as ibuprofen and paracetamol. 

While headache associated with vomiting is listed as a red flag in the NICE guidelines [17], the absence of other concerning features - such as positional headache, multiple episodes of vomiting and nausea, neck stiffness, neurological deficits, and an overall clinical presentation more suggestive of migraine or primary headache - led us to manage this patient’s case conservatively, warranting no further investigation. 

This approach is further supported by the American Academy of Neurology and the Child Neurology Society’s practice parameter, which states, “Obtaining a neuroimaging study on a routine basis is not indicated in children with recurrent headaches and a normal neurologic examination. Neuroimaging should be considered in children with an abnormal neurologic examination or other physical findings that suggest CNS disease” [18].

Context within the literature and case uniqueness

While uncommon, pediatric hypertensive encephalopathy presenting with seizures has been described in the literature. For instance, Sanford and Stein reported a three-year-old boy who presented to the ED with status epilepticus and was ultimately diagnosed with hypertensive encephalopathy [19]. In the study, initial neuroimaging (CT/MRI) did not immediately reveal the full extent of the pathology, as hallmark radiological findings such as vasogenic edema may lag behind symptoms [19]. This further stresses the importance of blood pressure management in pediatric patients who present with seizures. Imaging may support the diagnosis but should not delay recognition or treatment, since normal or non-specific early findings do not rule out hypertensive encephalopathy.

Nevertheless, our case adds insight into hypertensive emergencies in a region where epidemiological data on pediatric hypertension and related complications remain deficient. According to Almahmoud et al., data are insufficient regarding the prevalence of hypertension among adolescents in the Arab world, and little published work on the same topic exists [20], further highlighting the need to broaden clinicians' awareness in this geographical context.

## Conclusions

This case highlights the importance of blood pressure measurement, which should be routinely performed and carefully interpreted in all pediatric patients presenting to the ED, particularly those with seizures. Hypertensive encephalopathy and its complication, PRES, although rare, must remain important differentials in children with new-onset seizures or status epilepticus, and timely initiation of antihypertensive management is critical even before confirmatory neuroimaging is done.

Secondly, early multidisciplinary evaluation, including neuroimaging, echocardiography, and ophthalmological assessment, can help identify any end-organ involvement. Finally, pediatric patients with severe hypertension should also undergo thorough investigations for secondary causes and be closely followed by pediatric subspecialists to ensure long-term control and prevention of symptom recurrence.
